# Mission Himalaya: Exploring the Impact of a Supported High-Altitude Mountaineering Expedition on the Well-Being and Personal Development of UK Military Veterans

**DOI:** 10.3390/ijerph19095049

**Published:** 2022-04-21

**Authors:** Christopher William Philip Kay, Harriet Laura Wingfield, Jim McKenna

**Affiliations:** 1 Centre for Human Performance, Performance in Extreme Environments, Leeds Beckett University, Leeds LS1 3HE, UK; j.mckenna@leedsbeckett.ac.uk; 2 Social & Economic Research Institute, Sheffield Hallam University, Sheffield S1 1WB, UK; harriet.wingfield@student.shu.ac.uk

**Keywords:** psychological well-being, veterans, behaviour change, mental health, adventure therapy, recovery, health coaching, post-expedition growth, expedition, mountaineering, psychosocial development, self-determination theory

## Abstract

Meaningful, positive, emotional and challenging adventurous activities may generate personal growth or recovery from ill health or injury. In this study, we used a distinctive longitudinal and immersive research approach to explore the psychological impact of a high-altitude expedition to the Nepalese Himalaya on 10 (9 males) UK military veterans with longstanding well-being concerns. In the 12 months prior to the expedition, participants took part in three training weekends in the UK mountains. During the expedition, instructors—who were all experienced health coaches—facilitated reflective practices with the beneficiaries throughout, focusing on experiential transfer to day-to-day lives after the expedition. Follow-up interviews, conducted up to 18-months post-expedition, identified that the most desirable changes aligned with the three innate psychological needs of self-determination theory: autonomy, competence and relatedness. The routines established during the preparation stage and during the expedition itself activated a renewed energy for personal improvement. At 18 months post-expedition, the key changes reflected altered perspective, employment skills and work–life balance, increased physical activity and enhanced personal awareness and mindfulness. Importantly, supported by regular health coaching and focused on the transfer of learning, expeditions can activate meaningful long-term changes to the well-being and personal development of military veterans.

## 1. Introduction

Military personnel can experience circumstances during their time in service or as a part of their transition to civilian life that have a negative impact on their physical or mental health. A recent increase in service personnel reporting mental health concerns has identified that issues are not exclusive to those with deployment experience [1,2]. Notwithstanding, the prevalence of mental health problems is higher in deployed veterans [3]. Mental health problems are the second most prominent reason for medical discharge behind musculoskeletal disorders and injuries [4]. In the last 10 years, in the UK, medical discharges from the Army due to mental and behavioural disorders has increased from 15 to 33% [4].

The Parliamentary Defence Committee reported that some departing personnel are not adequately served by the system [5]. Many services that aim to support veterans stem from clinical models of recovery and continue to dominate rehabilitation services, focusing on symptom reduction and physical functioning [6]. Research has highlighted the need to explore additional and alternative approaches to recovery beyond conventional practice as an adjunct to the clinical services which “are not associated with hospitals, rehabilitation centres, or other clinical settings” [1]. One such area is sport and outdoor and adventurous physical activity (OAA), which is thought to complement mainstream practices by “facilitating a faster return to healthy levels of psychological functioning” [7].

The emerging literature examining the use of OAA programmes for veterans attributes such initiatives with some success in demonstrating improved well-being. This can be achieved through OAA provision, when delivered in a way that successfully provides an immersive experience, meets the bespoke needs of participants and is evidenced in an appropriate form [8]. Several studies have shown that conditions experienced on an expedition can result in improved psychological well-being [9,10,11,12]. However, few studies have examined the impact of expeditions for people adjusting to life-changing circumstances, such as sustaining career-ending physical injuries or the diagnosis of a trauma-related mental health disorder.

Only a few studies have reported on how short-term expeditions influence well-being benefits and these are limited by focusing on the immediate expedition, meaning that longer-term impacts are unclear [9,10,11,12]. Further, only one study has addressed the unique context of expeditions involving military veterans [13]. That study focused on the psychosocial impact of a mountaineering expedition on four individuals. The expedition influenced how individuals understood their own capabilities and reshaped their personal understanding of being a veteran, encouraging them to take more responsibility for their own recovery. Greer and Vin-Ravi [14] emphasised that more research is needed to establish the viability and appropriateness of expeditions to determine the durability and “real world” relevance to their outcomes.

In response, the aim of this study was to use a high-altitude expedition to the Himalaya to investigate the impacts on personal development and well-being that occurred amongst a small group of UK military veterans with a range of mental and physical health challenges. Further, given the pre-existing skills of the expedition staff, we aimed to activate expedition impacts by deploying health coaching, emphasising increased self-determination.

## 2. Methods

### 2.1. Study Design

To assess sustained positive impacts on health and well-being arising from participation in an OAA, semi-structured interviews were conducted with participants before, during and up to 18 months after a demanding month-long Himalayan mountaineering expedition. In this longitudinal study design, interviews with participants were carried out by a single individual researcher. Pre-expedition research was conducted to understand the existing changes participants wanted to make to their lives to improve their well-being and how they believed the expedition could facilitate this. The longitudinal element of this study design allowed the effects of this OAA intervention to be examined over an extended period of time, to assess the lasting effects of the expedition on the well-being and personal development of the military veterans.

### 2.2. The Expedition

Through an immersive expedition to Nepal, supplemented by three UK-based training weekends, the Mission Himalaya expedition was developed in line with the theoretical framework that underpins the delivery of developmental courses for veterans at the Battle Back Centre [15]. The expedition involved a 23-day trek in the Everest region of the Nepalese Himalaya, including an attempt to summit Mera Peak (6476 m/21,247 ft).

### 2.3. Theoretical Orientation of Health Coaching

Beyond exploring the impact of the expedition itself, this study also explored how self-determination coaching could amplify well-being benefits over a long-term period. Ryan and Deci [16] define well-being as “a complex concept, primarily concerned with optimal psychological experience and functioning”. As a key well-being theory, self-determination theory (SDT) is concerned with the motivation of behaviours, holding that positive growth, integrity and well-being rely on an individual’s basic psychological needs for autonomy, competence and relatedness [16]. Autonomy refers to the ability for an individual to make independent decisions and take control of one’s actions. Competence is associated with an individual successfully achieving mastery of activities and goals. Relatedness entails an intrinsic need to feel connectedness through both care of others and experiencing a sense of belonging. These three concepts provide practical actionable levers for any health coaches seeking to optimise intervention outcomes focused on psychological well-being.

### 2.4. Recruitment and Ethical Consent

The only inclusion criterion was to be a veteran member of the Mission Himalaya Expedition. Initially, all veteran expedition members were invited to participate in the study on a training weekend three months prior to the expedition. Prior to acceptance, all participants received a verbal briefing about the purpose of the study, reassurances about anonymity and confidentiality, made by the expedition researcher (CK). All agreed, giving a sample of nine males and one female.

Subsequently, participants were provided with an information sheet, via email, detailing the aims and methodology of the study and explaining in lay terms what would be expected of them if they agreed to participate. Potential participants were asked to take a minimum of 24 h before acknowledging that they had read and understood the participant information sheet after which time informed consent was obtained from those wishing to take part in the study through completion of an electronic consent form. Ethical approval for the study was obtained from the Leeds Beckett University Ethics Committee (50538).

### 2.5. Participants

All 10 participants were UK veterans of the armed forces, all of whom had faced personal, physical or mental health difficulties in their lives, some of whom were medically discharged from service. They were members of the Royal British Legion’s (TRBL) Mission Himalaya expedition to Nepal in 2018. None had trekked in the Himalaya before and only two had any mountaineering experience. The length of time since the participants had served ranged from 2 to 31 years, and participants were aged 29 to 62 years. Pseudonyms are used to maintain participants’ anonymity.

### 2.6. Health Coaching on a High-Altitude Mountaineering Expedition

The Mission Himalaya experience was developed from the five-day Battle Back courses that support UK veterans and recovering serving personnel. The courses use adaptive sport and adventurous activities as a context for personal growth and development. Staff operate in a health coaching capacity with the participants they support and host meaningful reflective practices to facilitate sustainable positive behaviour change [17].

This ethos was transferred to the development of the Mission Himalaya trip for the 10 veteran beneficiaries. All expedition staff were experienced Battle Back health coaching staff, meaning they were fully inducted into using the self-determination theory that characterises those courses, namely that of encouraging long-term behaviour change through the improvement of participants’ psychological needs of autonomy, competence and relatedness.

Three of the four expedition coaching staff were veterans themselves, with years of military experience, and all were experienced mountaineers who built close, positive working relationships with team members across the 12 months of expedition training prior to the expedition. The dual role of coach and mountaineering instructor led to a wide acceptance of their physical and emotional role in supporting the participants. Intentionally, the coaching staff worked specifically with two or three of the participants to develop a close, supportive relationship. They proactively engaged in reflective practices with their veterans at appropriate times on the expedition, on rest days and while at camp in the evenings, etc.

### 2.7. The Expedition Researcher

The expedition researcher (CK) was a climbing and mountaineering instructor. From three months prior to the expedition taking place, he took part in all aspects of the pre-expedition, training weekends, camping and hill walking with the participants, etc. He then participated fully in the expedition to Nepal, trekking with the participants, spending time with them on rest days and at camp. He also summited Mera Peak with one of the veterans and coaches. This immersion helped to build familiarity, trust and rapport and allowed for an in-depth account of what took place throughout the expedition. Creating the opportunity to build such a close relationship with the participants was intended to improve the likelihood of gathering meaningful and honest accounts of the participants’ experiences during the research interviews. Field notes, maintained throughout the expedition, detailed key events and participant concerns, personal development progress and challenges.

### 2.8. Data Collection

The researcher conducted semi-structured interviews with the 10 participants in two distinct phases. Phase one interviews were conducted before and during the expedition. Phase two interviews took place after the expedition over 6–18 months. Interviews involved the researcher using a list of topics or questions as a guide to cover relevant subjects during the discussion [18]. The use of semi-structured interviews allowed continuity between meetings and gave flexibility to the themes, encouraging a more relaxed discussion, aiming for rich and detailed data [19].

The Stages of Change model, which includes a series of five stages (pre-contemplation, contemplation, preparation, action and maintenance) that an individual will experience for behaviour transformation, was used as a guide to structure the phase one interviews [20]. Phase one interviews therefore focused on participants’ responses in terms of readiness to change. Conducting pre-expedition research aimed to understand what participants believed they could gain from the expedition and provided an individualised understanding of what success meant to everyone. Phase two interviews all took place via telephone, with a focus on the outcomes of the expedition and personal development of the participants.

### 2.9. Data Analysis

Semi-structured interviews were audio recorded, then transcribed and analysed using Braun and Clarke’s [21] six-step thematic analysis (TA) method. Researchers categorised the text data into codes, which were then grouped into coding sets based on key themes. This “inductive analysis” is a data-driven form of TA, involving the coding of data beyond constrictions of a pre-existing coding frame. The resulting flexibility allowed themes to emerge from the data. To avoid overlooking close details, NVivo software package was used in the coding process. Direct quotes were taken from the interview transcripts to illustrate the participants’ experiences within each key theme. To address the guiding framework of the health coaching underpinning the expedition experience, SDT concepts (autonomy, competence and relatedness) were also used as sensitising themes.

## 3. Results

### 3.1. Phase One—Before and during the Expedition

#### 3.1.1. Worthiness of Recovery Support

Unsurprisingly, the experiences of all 10 participants varied during and after their time in the armed forces. Some participants had been medically discharged, others were diagnosed with healthcare issues or injuries and some had no clinical diagnosis of illness or injury. Despite various struggles with their mental health, when posed with the opportunity to participate in the expedition, many of the veterans felt “*undeserving of being a participant*” (Ellis) or “*a little bit of a fraud*” (Sam). This was particularly common in cases where the participants had not sustained severe physical injuries but mental or moral injuries and considered themselves “*not a veteran that has been blown up*” (Ellis). When faced with the expedition application, several of the participants demonstrated high levels of self-doubt, stating how they had felt there was “*no point in applying*” (Ashley) and described having a “*definite lack of belief*” (Jo) that good things could come from the expedition.

The extent to which participants felt the expedition could facilitate positive changes in their lives varied from very little to complete belief that it would “*be a beneficial part of recovery*” (Ellis) and significantly benefit their well-being. Some of the participants also recognised that the expedition had the potential to benefit their physical health positively, enabling them to “*get a bit fitter*” and “*lose a bit of weight*” (Pat). The participants’ lack of self-confidence in relation to their eligibility for the expedition highlights their inability to take control of their own actions or make decisions and demonstrates a low level of autonomy and competence before the trip.

#### 3.1.2. What Did the Participants Want to Change in Their Lives?

##### General State of Mental Well-Being

The most common desired life change mentioned by the participants during the pre-trip interviews was in relation to improving their general mental well-being and personal development or growth. For differing reasons, the grounds behind leaving the armed forces had left many of the participants in a poor state of mental ill health or a “*bad place*” (Jo). Before the expedition, some participants described their personal situation and state of mental health negatively, for instance, as “*suffering terribly from social anxiety*” (Dan) and feeling “*tearful and stressed out*” (Ellis). One participant stated that they were “*really struggling with suicidal thoughts*” (Charlie) and another described themselves back then as “*a pretty negative person*” (Pat), going on to explain that they had been “*in therapy, trying to juggle different medications*” at that time. Others similarly recalled “*things breaking down at home… and that’s when I went off the rails*” (Jo). Following significant personal setbacks experienced around the time of being medically discharged from the armed forces, one participant explained wanting to return to “*operating at full potential*” (Ellis) following the trip. After undertaking training weekends for the expedition, the participants began to recognise the potential “*amount of time to reflect on stuff*” (Elliot) the trip would provide them with and the satisfaction they could feel from completing tasks and goals during the trip. They deduced that these tasks could enable them to “*come back in a mentally better place… with something concrete and say yeah I have done this or done that*” (Charlie). In speaking generally about their mental well-being, the majority of the participants hoped that the experiences on the trip would act as “*a period of growth*” (Ellis) and enable them to feel “*mentally enriched*” (Sam) on their return, with a rejuvenated sense of meaning and purpose. The descriptions of the participants’ personal situations before the expeditions demonstrated that their mental state of well-being was generally quite negative. They recognised that by achieving goals associated with the trip, they would feel more satisfaction in life, which would be a result of increased competence.

#### 3.1.3. Communication and Relationships with Others

In addition to improved general mental well-being, many of the participants expressed desires for the expedition to enable them with the skills and experiences to improve their communication and relationships with others. Before the expedition, a number of the participants described their struggles in tolerating others or “*maintaining healthy working relationships with people*” (Jo) and being “*able to work well in a team*” (Jamie). The desire to develop more empathy for others was commonly discussed amongst multiple participants, recognising how this could benefit them “*generally through life and day to day situations*” (Jamie) and reduce the risk of them getting angry. Jamie, in particular, acknowledged how, before the expedition, his angry outbursts had resulted in violent exchanges which could have resulted in imprisonment, recognising that significant changes needed to be made to avoid recurrence of this situation. In addition to improving tolerance and team-bonding skills, some of the participants recognised that the expedition could facilitate them in “*developing a social network of friends*” (Frankie) and enable them to build relationships with other veterans in a similar situation to themselves. By spending an extended period of time with a variety of different individuals on the expedition, the participants noted that their communication skills and team-working ability would be tested. One participant commented in depth on how they imagined the expedition may help to improve their ability to interact with others:

“*It will put me in a challenging position where I might be tired and stressed, but let’s take that back to a workplace where I might be under pressure […] translate that to being absolutely knackered at altitude and being sick and stressed; and the relationships might be under strain in the group. The expedition will replicate the most extreme circumstances where you will have to kind of really apply that team ethic and understanding of other people and tolerance.”*

(Jo)

Expressing a distinct need for improvement in their ability to interact with others and build stronger relationships through more tolerance exemplified the participants’ lack of satisfaction with their basic psychological need for relatedness.

#### 3.1.4. Employment

Many participants talked about wanting to improve their employment situation, improve their job satisfaction, find work or take up a more prolific job to increase their earnings to support themselves and their family. Many participants noted their struggles in settling into a new career since leaving the armed forces. Before the trip, participants described feeling “fed up with the same s*** every day… and hated every minute” (Ashley) of their working lives. Many of the participants displayed a level of dissatisfaction with their employment situation, for instance, Frankie expressed not having *“a career as such yet”.* Frankie did however recognise that the expedition could “*open a few doors for myself and maybe others… show me a new world and you never know a future career*”. Four of the participants discussed wanting to use the expedition to improve their mountain skills and subsequently enhance career opportunities in the outdoor sector or as a future leader on trips such as Mission Himalaya to support other military veterans. Ellis noted that the expedition would be “*a really fantastic experience personally to get something great out of it… and see people doing stuff that I would like to do at some point*”. The expedition was viewed by the participants as a potential opportunity to unlock knowledge about “*going to the Himalayas and being on expeditions with a group… all experience that goes towards my CV*” (Elliot).

Changes regarding their ability to secure employment was key for the majority of the participants. They maintained that if changes such as improving their ability to “*work in a challenging professional manner again and… make an impact*” (Ellis) were not made, they risked being left not working to their full potential or settling on something they would be dissatisfied with. Charlie stated that by going on the expedition and learning “*what limitations I have*” would enable a better understanding of “*what kind of career I could be best suited to*”. By increasing their skills and confidence levels during the expedition, the participants foresaw that Mission Himalaya could help their future job applications, such as Ashley in “*applying for the Fire Brigade next year*”. Increasing their confidence to independently undertake tasks associated with employment had the potential to address the low levels of competence that many of the participants displayed whilst also enabling them to feel a greater sense of purpose.

#### 3.1.5. Physical Health and Routine

Physical health and fitness were also a key theme of the phase one interviews. Every participant mentioned physical health and fitness in relation to aspects of their life they wanted to change. For some, this was wanting to “*get back to who I used to be, physically… and use the expedition as a way of driving me back into the outdoors environment*” (Charlie). Many of the participants recalled a previously high level of physical fitness from their days in the armed forces, describing how, since becoming a military veteran, they had “*let themselves go”* (Jo) or “*got quite fat… and sort of been up and down with weight*” (Frankie). During the discussions about their physical health before the trip, the participants discussed in depth how their poor state of mental health had impacted their physical health and fitness or motivation to go out and exercise:

*“A typical day was getting up around four o’clock every morning, erm, sorting the dog out if he was at home and then going to work. Doing a twelve to fifteen hours day lorry driving and then if I wasn’t sleeping out in the cab I was coming home and was basically chilling out or lying around on the settee before bed because I was knackered.”*

(Ashley)

In the lead up to the expedition, with the motivation of the trip and training weekends, many of the participants recognised the positive mental health benefits that resulted from them becoming more active again, stating how they “*felt happy… and your mind feels much clearer*” (Ashley) after exercise or outdoor physical activity. Most of the participants noted that even at this early-stage interview, having the motivation to train for the expedition helped them “*lose weight*” (Pat) and become “*so active*” (Frankie) which gave them “*more get up and go… and confidence to grow by the day*” (Jo). Many participants linked the aspiration to improve their fitness levels with their desire to create more routine in their lives. They surmised that the “*regular routine and getting up early*” (Jamie) during the four-week expedition would re-introduce them into the positive rhythm of having a daily routine and instil habits they wanted to maintain “*doing when I get home*” (Dan). Charlie stated that a “*mindset which involves getting up and doing physical activity is going to improve my physical and mental health*”. The process of preparing for the expedition clearly aided the participants in an improved sense of confidence, physical fitness and overall mental well-being, much of which they “*largely attributed to being able to use the training weekends as short-term goal setting for fitness*” (Jo). By utilising the expedition to regain ability in controlling their own behaviours, the participants had the opportunity to improve their autonomy.

#### 3.1.6. Readiness to Make Change

When asked if they felt ready to use the expedition to facilitate making the aforementioned positive changes to their lives, the majority of the participants demonstrated their commitment in feeling ready for change. The intensity or level of readiness for change ranged from some individuals reporting feeling slightly less confident about their ability to make these changes, to others feeling *“100% committed to those changes*” (Jo). Despite some concerns, the majority of the participants were confident in feeling “*beyond ready… with no hesitation*” (Sam) and many noted that the risk of not making these changes could result in lower moods, reduced motivation and well-being, described by Frankie as a *“spiral down into depression again I suppose, just not going anywhere and just going around in circles*”.

The findings from the interviews before the expedition highlighted the need for improvements to the levels of autonomy, competence and relatedness for the participants, who had all struggled with their mental health to varying extents and in multiple areas of their lives, following their exit from the military. The findings from the phase one interviews highlight the desire and readiness for change in the majority of the participants.

### 3.2. Phase Two—6–18 Months after the Expedition

The findings from the phase two interviews are grouped based on the topic areas that emerged from the conversations. This section will present the evidence for the extent of attribution, expressed by participants, of the transfer of the experience to their day-to-day lives.

#### 3.2.1. Altered Perspective

A key theme to emerge from the follow-up interviews was the altered perspectives of many participants, which they experienced following their involvement in the expedition. The key factors for this change in outlook were identified as emerging via mental and physical challenge, cultural exposure and learning about other people’s recovery journey. Physical and mental challenges were an almost daily occurrence on the expedition, which involved being at an altitude of between 3000 and 5000 m for most of the trek. The nature of this high-altitude expedition additionally involved disturbed sleep and emotional strains of working as a group. For many, the expedition was extremely challenging, for instance, Jamie described it as “*mentally and physically the hardest thing I have done*”. Multiple participants reported that the tough moments they faced and the challenges they overcame led to a shift in their perspective of what was possible for them, both physically and mentally:

*“If you overcome adversity in your life, then you end up being able to cope with it... I am not worried about panicking so much because I know I can control it, but I am also not worried about saying no and saying actually I don’t want to do it... I don’t want to push myself beyond that... whether that be exercise or whether that be at work.”*

(Pat)

In addition to the challenges that altered their perspectives, many participants spoke about the way in which the exposure to “*Nepal as a whole country and the people*” (Sam) influenced their perspective on their own lives, their ways of thinking and their ability to be more content with their living circumstances:

*“If everyone could go to Nepal and sort of see how it is actually possible to be happy with very much less than what we have over here […] so much stuff that we have and do is really unnecessary. I certainly look at things differently. I think we all appreciated that to a degree at the end, having the perspective that I have from Nepal, it’s really helped.”*

(Charlie)

Learning about the recovery journey of others emerged as another way in which the perspectives of the participants were altered. The time away in Nepal presented multiple occasions in which the participants could interact with other veterans in smaller sub-groups and have open conversations, sharing experiences. For many, understanding “*the mental health issues and trauma some people have been through*” (Sam) helped to influence their perspective on their own situation and enabled these participants to “*draw strength from how others dealt with it*” (Charlie). Impacting the perspective of the participants and potentially influencing their outlook or purpose in life aligns with improved well-being, undoubtedly initiated by their experience on the expedition.

#### 3.2.2. Employment Skills and Work Life Balance

In discussing some of the changes made in their lives as a result of the expedition, over half of the participants discussed substantial changes in their careers. One participant in particular, Pat, a veteran of the Royal Army Medical Corps, described a significant change in their employment situation. Before the expedition, Pat was unable to work and was in a state described by themself as very “*negative*”, undergoing therapy and taking multiple medications. Pat had been advised to seek employment outside of medicine, since attempts to return to work had been so unsuccessful. When exploring Pat’s perspective of attribution, Pat described how the expedition had enabled a “*change in where I was positioned*” which helped in being “*more confident in my ability to make choices*” and “*taking on more responsibility*”. The expedition also helped Pat with learning to control anxiety attacks, providing opportunities to “*practice breathing and controlling it*”. Pat attributed the expedition as partially responsible for the regained confidence in themself and their ability to work, stating, “*I don’t know if I would have ended up at this point had I not gone on an expedition. I don’t know if I would have ended up back at work*”. The confidence in decision making described by Pat, which resulted from the experiences of the expedition and translated into the participants’ daily work or lives, was supported by others who also felt they had “*more confidence*” (Jamie) in their “*own decision making ability*” (Charlie). Having a higher level of self-belief with regard to making independent decisions and feeling in control of one’s behaviours and destiny illustrates the increased autonomy that resulted from the expedition and subsequently improved the participants’ mental state of well-being.

Multiple other participants found they could attribute the expedition to helping them have a new perspective on the importance of their work style, which emerged when discussing the changes to their careers following the expedition. Ashley, for instance, fulfilled a long-term goal of “*working for The Fire Service*”*,* despite the struggles in applying for this position before the expedition. Other participants attributed the expedition to providing them with “*more structure and stuff to aim for*” (Charlie) in relation to financial security and employment. Having the ability to master tasks, such as securing new job roles and achieve goals linked to financial security, represents improved satisfaction with the basic psychological need for competence in these participants following the expedition.

#### 3.2.3. Relationships with Others

Two key relationships on the expedition for the participants were those with the staff and those with the other team members. The influence both of those had on subsequent relationships with friends, family members and the community after the expedition was commented on widely by the participants. The verbal encouragement and support received by the staff members on the expedition was described by the participants as “*a massive pick-me-up*” (Charlie), with many mentioning how beneficial they had found being able to have multiple ongoing one-to-one conversations with staff members. Pat described these chats as useful to “*talk through things and focus on the smaller things and managing me*”. In addition to the support from the expedition staff, all the participants mentioned the “*camaraderie and togetherness of the group… the strengths and the weaknesses that we pulled each other through, the good and the bad times*” (Sam). The strength in the relationships the participants built amongst one another were enabled through the “*shared experience*” (Charlie) and the nature of a high-altitude expedition. For instance, sharing a tent with another team member on the expedition enabled the participants to “*build those relationships and bonds*” (Ellis) that were perhaps less easy to achieve back home. The unique nature of spending three weeks walking with others enabled the veterans to “*speak to people a lot more and you got to know them a lot deeper as a person… it was so easy to speak about very deep things, without people being afraid*” (Sam). Charlie confirmed the depth of these relationships through the distinctive characteristics of an expedition, stating that going to Nepal was a:

*“… unique experience for those involved, it’s quite a long way different from the, sort of, day to day experiences of the vast majority of people. I think all of those things sum up together to make it a much stronger bonding experience.”*

(Charlie)

Some of the participants felt that the relationships they built with others on the expedition motivated them in wanting to help others when they got home and “*put something back into the veterans community*” (Pat). The importance of “*maintaining those contacts… and being amongst others, being able to talk freely and more openly*” (Charlie) was important to many participants, who used the expedition to build strong relationships with individuals that became “*lifelong friends*” (Pat). Upon returning to their partners and families after the expedition, some of the participants discussed how their experiences and the personal development they had undergone throughout the expedition and afterwards had impacted their existing relationships at home:

*“When you come back, I certainly made more of an effort to do things together, which probably I did before, but not as much. And we make time for conversation now, and we sit outside and just talk a lot more. So yeah, so I certainly appreciate my partner a lot more.”*

(Sam)

Increasing the ability of the participants to feel a sense of attachment and belonging to others, during and after the trip, highlights the impact a high-altitude expedition of this kind can have on the relatedness of military veterans.

#### 3.2.4. Behaviour Change: Physical and Mental Health

The physically demanding nature of the expedition had a generally positive influence on the activity levels and physical health of the participants. One participant described the trip to Nepal as significant because “*it revalidated me physically*” (Charlie) or encouraged the participants to “*start doing a bit more training*” (Jamie). Others described how they had maintained high levels of physical activity following the trip to Nepal and attributed their motivation to the expedition:

*“I’m still probably doing somewhere between 60 and 70 km a week, walking and I don’t lack motivation to do it... honestly, I don’t think I would have the mental strength and the mental thinking, without doing what I was lucky enough to do on the expedition.”*

(Sam)

These reported behavioural changes resulting from expedition participation indicate improvements in the participants’ confidence to perform physical activities or tasks. All the participants made statements suggesting an improvement in their satisfaction of their psychological need to feel competent. In addition to the longer-term physical health benefits, many participants discussed the changes to their behaviours associated with mental health, such as to their enhanced personal awareness following the trip. The intentional focus that was put on personal development by the coaching staff developed participants’ reflective practice after the expedition and enabled them to “*function better*” (Jo). Many of the participants described how their “*thinking process is now totally different to how it used to be… the whole thing made me a better thinker*” (Sam). Being a “better thinker” translated into the participants describing how they felt calmer and more relaxed in difficult situations and like they can now *“deal with stuff much better*” (Charlie). The experiences on the expedition enhanced the ability for individuals to feel independently in control of their mental health, reflecting improved autonomy.

### 3.3. Summary of Results

In the interviews before and during the expedition, all participants alluded to a lack of fulfilment of the basic psychological needs of autonomy, competence or relatedness. For some, it was in expressing their lack of worthiness for support, intolerance of other people, displeasure with their work circumstances or lack of self-belief. In the phase two interviews, all the participants had shifted to describing improvements in aspects of their life, behaviour and thinking that align with the basic psychological needs from the SDT. Interview evidence, drawn from multiple timepoints, indicates the ongoing and sustained change in well-being and personal development. The three needs and their alignment with the resultant themes are identified and summarised in Table 1.

## 4. Discussion

### 4.1. Summary of Key Findings

The principle finding from this study, involving 18 months of engagement with members of Mission Himalaya, was that the experience of a high-altitude expedition involving intentional health coaching can facilitate long-term meaningful change for participants. This prolonged and intentionally designed experience helped participants to detach themselves from their home contexts, to focus on the context of the expedition, and as a result, to regard themselves—and describe themselves—“with more distance”. Most participants had successfully begun to see themselves as a “project” and were working to become more successful back at home. Post-expedition, many had successfully adopted new behaviours, such as regular physical activity, that many in their home contexts continued to find difficult.

The findings from the pre-expedition interviews highlighted the deep desire to improve levels of autonomy, competence and relatedness. All participants had experienced some struggle with mental health—to varying extents and in multiple areas of their lives—after leaving the military. The findings also highlighted that a majority desire and have a readiness for change. Describing their histories, pre-expedition, many discussed lacking in self-belief or the ability to make independent decisions over their actions. This extended to not feeling worthwhile enough to apply for recovery support and/or to the expedition. Selection for the expedition was a powerful signal of redirection. Future expeditions should make the application process as welcoming as possible.

Early interviews highlighted the many areas where participants wanted to change; interviews afforded an opportunity to stand back and reflect on personal progress desires. These needs underlined the relevance of using the SDT to ground our health coaching approach. Equally, the scale of change that had been transferred to life back home confirms the utility of the transfer of learning approaches used.

Shortfalls in relatedness were often linked to poor communication skills, limited empathy for others and a weak sense of belonging. Competence was undermined by widespread difficulties with employment: some participants had failed to re-enter employment after leaving the military, while others lacked confidence in their work environments. It was common that physical activity had reduced upon leaving the military; lack of routine was commonly a problem here. For some, the preparatory training weekends mandated additional physical activity, and this helped to re-establish motivation and structure. Many participants also hoped the routines of the expedition would re-establish lost routines or behaviours back home, leading to improved autonomy.

The majority of the participants demonstrated a strong commitment and readiness to make changes to their lives through the Mission Himalaya expedition. Those with a greater receptiveness for change tended to experience more significant effects and changes following the expedition. The results of this study therefore support the observation of Smith et al. [11] that participants’ agreeableness and openness play a key part in post-expedition growth. Despite this, one participant in particular (Pat), who expressed less readiness for change and belief the expedition may facilitate this, subsequently reported significant positive changes in their occupational circumstances, which were partly attributed to the expedition, following a “*light bulb*” (Pat) experience whilst being extremely challenged at high altitude. This illustrates that openness and readiness to change does not necessarily always correlate to the total possible change.

The findings from the follow-up interviews that took place in the 18-month post-expedition period evidence the changes that were made by the participants and to what extent they attributed the changes to the expedition. The majority of the participants discussed an altered perspective on their own lives. This change in their outlook was attributed to a number of factors. First, undertaking challenging tasks during the expedition increased their confidence and ability to undertake tasks back home. Successful mastery of tasks in specific environments has the potential to improve the self-efficacy and competence of an individual [22]. Second, cultural exposure to Nepalese people who had significantly lower standards of living and money, compared with the participants, enabled them to feel more grateful for their own lives. Tourism literature widely supports the notion that exposure to host cultures and subsequent reflection is often a mechanism for transformation [23]. The participants supported this in reporting a greater sense of meaning and purpose in their own lives after these cultural experiences on the trip. Additionally, learning about the recovery journey of others stimulated reflective thought in the participants about their own lives, compared to the problems faced by others similar to themselves. This also influenced their perspectives and provided them with a greater sense of meaning, contributing to their ongoing personal development and enhanced well-being.

The interview conversations following the trip highlighted multiple occurrences in which the participants attributed involvement in the expedition to the changes they had managed to achieve in relation to their employment or job satisfaction. Many participants reported an improved ability to master tasks such as job applications, which they had not been able to undertake before the expedition, demonstrating an improvement in their basic psychological need for competence. Building strong relationships with the staff and other military veterans during the expedition enabled many of the participants to improve their interactions with friends, family and colleagues back home afterwards. The specific characteristics of a challenging OAA intervention of this kind, such as sharing tents, multiple days spent walking and time away with the expedition team, were all found to contribute towards the improvement of the participants’ relatedness. The physically demanding nature of the expedition had numerous positive effects on the physical health of the participants, who reported training more in preparation for and as a result of the expedition. In line with positive physical health changes, many participants discussed the changes to their mental health after the expedition. This improvement to the thinking processes and mental strength of the participants was aided through the specific coaching of the staff who intentionally focused on personal development during the trip.

The findings demonstrate that Mission Himalaya is another development in the application of adventurous activities to positively influence behaviour change and the well-being of participants. Previous studies to examine the use of OAA interventions with military veterans were cross-sectional in nature and therefore, despite their results confirming the positive impacts on the personal development and well-being of veterans, the studies were unable to observe whether these findings had a longer-term effect [13]. This research addresses the shortfalls of previous research studies and is the first study to follow the participants longitudinally. The results demonstrate that the observed positive impact of the expedition on the lives of the veterans persisted over the course of the study (up to 18 months after the expedition). The results of this study address Greer and Vin-Ravi’s [14] request for future OAA intervention processes and mechanisms that lead to positive outcomes to be better explained by providing a deeper insight into the ways in which this expedition facilitated enhanced well-being and personal development. Additionally, an improved understanding of life enrichment for veterans through the challenges presented by a meaningful goal, such as an expedition, could illuminate longer-term understanding and better knowledge surrounding the transition to civilian life as well as personal development [14]. This study has enhanced our understanding of the influence that challenging expedition experiences can have on participants’ lives to an extent that has not previously been achieved.

### 4.2. Strengths and Limitations

Many expeditions have previously failed to collect satisfactory data sets regarding participant well-being [24]. The resulting database has been dominated by low compliance, short expeditions, fee-paying civilian participants and a lack of longitudinal research periods. By embedding a researcher throughout the preparatory, expedition and post-expedition periods, the study is unique. Developing the delivery to be theory-guided and to address transfer of learning to ensure prolonged impacts makes it totally unique. As a result, our study represents an unrivalled database, generated from ten UK veterans across 18 months of qualitative data gathering. Participants will continue to be invited to contribute follow-up interviews for five years, until November 2023.

A small number of programmes, similar to Battle Back’s Mission Himalaya expedition, exist in other countries, such as the USA, where veterans from recent wars reside. Some of these programmes are similar in nature to Mission Himalaya, with the use of OAA to improve the lives of military veterans. Examples of these similar programmes in the USA include the Warrior Hike Program (WHP) and “Adventure Not War”. The WHP consists of a 6-month hike of the Appalachian Trail, aiming to provide a positive therapeutic effect by immersing participants in the natural environment [25]. Adventure Not War took military veterans back to Iraq for a mountaineering expedition, aiming to empower them to reclaim their lives despite their history with the country [26]. Although similar to Mission Himalaya in the context of using OAA to improve the well-being and development of military veterans, these expeditions differ in their lack of longitudinal investigation of the lasting effects on the veterans, a strength of the Mission Himalaya study.

Limitations include the significant investment of time and resources and being able to participate as an expedition member. This is likely to have built the rapport and respect that contributed to the high-retention rate in the follow-up interviews. The overall process is unlikely to be a viable research method and/or process for researchers who are not also mountaineering instructors.

An additional limitation of the study includes the gender balance of the sample. The predominance of males to females in the study sample is however reflective of the current UK Armed Forces which comprises 11% females [27]. Future studies could actively recruit more females.

An important realisation emerged through follow-up data collection; a return to normal domestic life in the UK left some members missing the social support, the physical challenges and the personal attention of the expedition. This left them framing the highs of the expedition with the lows of feeling let down when back in the UK. Expedition funders and organisers must ensure appropriate support is put in place to help participants manage post expedition “come down”.

### 4.3. Future Application

The study confirmed that positive health coaching can support participants in a high-altitude expedition. By intentionally including health coaching that accentuated “transfer of learning”, participants became skilled in applying their in-expedition learning to their day-to-day lives after the trip. This health coaching approach is suited to the expedition context as it is a principle-guided technique, meaning it can fit within the opportunities that emerge in the moment and/or unexpectedly. This research has useful transferable potential to provide a future template for expeditions centred on recovery and personal development. Practically, organisers of future OAA interventions could apply this knowledge to shorter expeditions in less extreme environments or destinations.

## 5. Conclusions

To our knowledge, this is the first study to research the long-term influence of participating in a high-altitude trekking expedition that involves health coaching on the lives of UK military veterans. The expedition itself is also believed to be the first of its kind. Our findings support the prolonged use of health coaching, focused on self-determination, to encourage experiential transfer back into what had often been “troubled” daily lives. The intentional blend of health coaching into a prolonged expedition was recognised as helping to make this a meaningful, long-term positive influence for these participants, beyond the expedition itself. In a mutually reinforcing way, the delivery approach and research style can be replicated in subsequent initiatives to positively influence the lives of participants.

## Figures and Tables

**Table 1:** Alignment of the desired life changes prior to the expedition and subsequent reported changes by the participants with the three basic psychological needs from SDT.

	Desired Behaviour Changes Prior to the Expedition					Reported Behaviour Change after the Expedition			
**Autonomy**	Gaining better control of their minds and thoughts	Having more routine and structure in their lives	To be liberated from external pressures		**Autonomy**	Altered perspective through the challenge of the expedition	Adjusting work–life balance and gaining routine	Improved self-worth and confidence in decision making	Personal awareness and increased mindfulness
**Competence**	Reducing a busy lifestyle and addressing issues with their occupation	Desire to progress in their careers	Improving physical health and fitness	Have an opportunity to succeed in a challenge	**Competence**	Altered perspective through the challenge of the expedition	Employment skills	Improved health and physical activity levels	
**Relatedness**	Improving relationships with others, family, work colleagues, etc.	Sense of belonging, connectedness with others	Regaining dignity	Being more understanding of others circumstances	**Relatedness**	Altered perspective through cultural tourism	Altered perspective through learning about other people’s recovery journey	Relationships with others	

## Data Availability

The data presented in this study are available on request from the corresponding author.

## References

[1] Dustin D., Bricker N., Arave J., Wall W. (2011). The promise of river running as a therapeutic medium for veterans coping with post-traumatic stress disorder. Ther. Recreat. J..

[2] Ministry of Defence (2018). UK Armed Forces Mental Health: Annual Summary and Trends over Time, 2007/08–2017/18.

[3] Phillips D., Marcinkiewicz A., Wishart R., Forsyth E., Nguyen A., Lynch-Huggins S., Gogescu F., Gilbert A., Sokratis D., Vojtkova M. (2020). The Mental Health Needs of Serving and Ex-Service Personnel: A Systematic Review.

[4] Ministry of Defence (2020). UK Armed Forces Mental Health: Annual Summary & Trends over Time, 2007/08–2019/20.

[5] House of Commons Defence Committee (2019). Mental Health and the Armed Forces, Part Two: The Provision of Care, Eleventh Report of Session 2017–19.

[6] Samele C. (2013). The Mental Health of Serving and Ex-Service Personnel: A Review of the Evidence and Perspectives of Key Stakeholders.

[7] Caddick N., Smith B. (2014). The impact of sport and physical activity on the well-being of combat veterans: A systematic review. Psychol. Sport Exerc..

[8] Coon J.T., Boddy K., Stein K., Whear R., Barton J., Depledge M.H. (2011). Does participating in physical activity in outdoor natural environments have a greater effect on physical and mental wellbeing than physical activity indoors? A systematic review. Environ. Sci. Technol..

[9] Allison P., Martindale R., Stott T., Gray S., Nash C., Fraser K., Wang J. (2018). The value of participating in British exploring society expeditions: A three year multi-cohort study. AUC Kinanthropologica.

[10] Michael M., Johannes M. (2016). Mental health benefits of outdoor adventures: Results from two pilot studies. J. Adolesc..

[11] Smith N., Kinnafick F., Cooley S.J., Sandal G.M. (2017). Reported Growth Following Mountaineering Expeditions: The Role of Personality and Perceived Stress. Environ. Behav..

[12] Stott T., Allison P., Wald K.v., Fakunle O. (2016). Exploring factors influencing outcomes of a five-week youth expedition in the Himalayas using the sail training programme self-assessment toolkit. AUC Kinanthropologica.

[13] Burke S.M., Utley A. (2013). Climbing towards recovery: Investigating physically injured combat veterans’ psychosocial response to scaling Mt. Kilimanjaro. Disabil. Rehabil..

[14] Greer M., Vin-Raviv N. (2019). Outdoor-Based Therapeutic Recreation Programs Among Military Veterans with Posttraumatic Stress Disorder: Assessing the Evidence. Mil. Behav. Health.

[15] Deci E.L., Ryan R.M. (2000). The “what” and “why” of goal pursuits: Human needs and the self-determination of behavior. Psychol. Inq..

[16] Ryan R.M., Deci E.L. (2000). Self-determination theory and the facilitation of intrinsic motivation, social development, and well-being. Am. Psychol..

[17] Frates E.P., Moore M.A., Lopez C.N., McMahon G.T. (2011). Coaching for Behavior Change in Physiatry. Am. J. Phys. Med. Rehabil..

[18] Kallio H., Pietilä A.M., Johnson M., Kangasniemi M. (2016). Systematic methodological review: Developing a framework for a qualitative semi-structured interview guide. J. Adv. Nurs..

[19] Roulston K., Choi M. (2018). The SAGE Handbook of Qualitative Data Collection.

[20] Krampe H., Salz A.-L., Kerper L.F., Krannich A., Schnell T., Wernecke K.-D., Spies C.D. (2017). Readiness to change and therapy outcomes of an innovative psychotherapy program for surgical patients: Results from a randomized controlled trial. BMC Psychiatry.

[21] Braun V., Clarke V. (2006). Using thematic analysis in psychology. Qual. Res. Psychol..

[22] Jackson B., Beauchamp M.R., Dimmock J.A. (2020). Efficacy Beliefs in Physical Activity Settings. Handbook of Sport Psychology.

[23] Van Winkle C.M., Lagay K. (2012). Learning during tourism: The experience of learning from the tourist’s perspective. Stud. Contin. Educ..

[24] Wetherill N., Taylor N., Hennis S., Barker Z., Stephenson J., Montagne S. Ice Maidens Expedition. Proceedings of the Performance in Extreme Environments Leeds Beckett University.

[25] Dietrich Z.C., Joye S.W., Garcia J.A. (2015). Natural Medicine: Wilderness Experience Outcomes for Combat Veterans. J. Exp. Educ..

[26] Brymer E., Rogerson M., Barton J. (2021). Nature and Health: Physical Activity in Nature.

[27] Ministry of Defence The UK Armed Forces Biannual Diversity Statistics. https://www.gov.uk/government/statistics/uk-armed-forces-biannual-diversity-statistics-2021/uk-armed-forces-biannual-diversity-statistics-1-april-2021.

